# Plasma 25-Hydroxy Vitamin D Is Not Associated with Either Cognitive Function or Academic Performance in Adolescents

**DOI:** 10.3390/nu10091197

**Published:** 2018-09-01

**Authors:** Abdur Rahman, Abdullah Al-Taiar, Lemia Shaban, Reem Al-Sabah, Anwar Al-Harbi, Olusegun Mojiminiyi

**Affiliations:** 1 Department of Food Science and Nutrition, College of Life Sciences, Kuwait University, Box 5969, Safat 13060, Kuwait; dr.lemiashaban@gmail.com (L.S.); a.n_a.m@hotmail.com (A.A.-H.); 2 Department of Community Medicine and Behavioural Sciences, Faculty of Medicine, Kuwait University, Box 24923, Safat 13110, Kuwait; altaiar@hsc.edu.kw (A.A.-T.); reem1@hsc.edu.kw (R.A.-S.); 3 Department of Pathology, Faculty of Medicine, Kuwait University, Box 24923, Safat 13110, Kuwait; segunade@yahoo.com

**Keywords:** 25-hydroxyvitamin D, cognitive function, adolescents, school performance, Raven’s Stranded Progressive Matrices

## Abstract

Several observational studies have reported an association between low levels of vitamin D (VD) and poor cognition in adults, but there is a paucity of data on such an association in adolescents. We investigated the association between VD and cognitive function or academic achievement among 1370 adolescents, who were selected from public middle schools in Kuwait, using stratified multistage cluster random sampling with probability proportional to size. Plasma 25-hydroxy VD (25-OH-D) was measured using liquid chromatography-tandem mass spectrometry (LC-MS/MS). An age-adjusted standard score (ASC), calculated from Raven’s Standard Progressive Matrices test, was used to evaluate cognitive function; academic achievements were extracted from the schools’ records. Data on various covariates were collected from the parents through a self-administered questionnaire and from the adolescents using face-to-face interviews. 25-OH-D was weakly correlated positively with ASC (ρ = 0.06; *p* = 0.04). Univariable linear regression analysis showed an association between 25-OH-D categories and ASC after adjusting for gender, but adjusting for parental education was sufficient to explain this association. Multivariable analysis showed no association between 25-OH-D and ASC after adjusting for potential confounders whether 25-OH-D was fitted as a continuous variable (*p* = 0.73), a variable that is categorized by acceptable cutoff points (*p* = 0.48), or categorized into quartiles (*p* = 0.88). Similarly, 25-OH-D was not associated with academic performance. We conclude that 25-OH-D is associated with neither cognitive function nor academic performance in adolescents.

## 1. Introduction

Low levels of serum vitamin D (VD) has become a global concern in all age groups, ethnicities, and geographical locations [1]. It is estimated that over 80% of the population worldwide has either insufficient or deficient levels of VD [2]. Such a high prevalence worldwide has led to a debate on VD levels that should be considered deficient or insufficient given the fact that most of those described as VD deficient do not suffer from obvious adverse health consequences [3,4,5]. It is worth noting that different organizations use different cutoff points to define VD deficiency and insufficiency, which may result in a large variation in VD status between different settings [6]. Low levels of serum VD is even more common among children and adolescents, particularly girls in some settings [7,8].

In addition to its well-established musculoskeletal effects, VD is hypothesized to regulate several other physiological functions as well. These include cardiovascular health, glucose homeostasis, immunity, and protection from cancer and neurodegenerative diseases [9]. However, a causal relationship between VD deficiency and these disease conditions has not been clearly demonstrated; and the evidence for the impact of VD supplementation from randomized controlled trials (RCTs) on the risk of these conditions is not conclusive. In fact, several RCTs have not demonstrated a beneficial effect in many of these disease conditions [10,11,12,13].

The role of VD in brain development and cognition has become of great interest in recent years. The active form of VD (1,25-dihydroxyvitamin D) is suggested to be involved not only in early brain development but also in adult brain function [14]. VD receptors (VDR) and metabolizing enzymes have been found in several brain areas that are involved in learning and memory in both humans and animals [15,16,17,18]. Thus, the ability of the brain to synthesize the active form of VD locally and the presence of VDR suggest that VD is involved in the development and/or normal functioning of the brain [19,20].

It has been suggested that VD may support neurological development during the prenatal period, increase cognitive abilities, and enhance cognitive reserve during childhood and adolescence, and prevent cognitive loss in older age [21]. A recent literature review of 26 observational studies and three RCTs supported the beneficial effects of VD on cognitive function among older adults based on observational studies but not RCTs [22]. However, studies that investigated the association between VD and cognitive function among adolescents are rare in the literature. Only two studies [23,24], both based on the National Health and Nutrition Examination Survey III (NHANES III) data, have addressed this issue and reported no association between VD and cognitive function [23,24]. 

The association of VD with cognitive function has different interpretations in children compared to adults or older adults. In the latter, the association may suggest that VD helps preserve cognitive function, whereas in children the association may suggest that VD assists in cognitive development [23]. Furthermore, it is sensible to study the relationship between VD and cognitive function in children and adolescents because it will not be confounded by negative health habits, like smoking and drinking, which are less common in this age group when compared to adults [23]. Cognitive function during adolescence or childhood is extremely important because it is associated with various health outcomes in adulthood [25,26], and if a causal relationship between VD and cognitive function is established at this age, there will be a potential to correct the problem early on, and later life adverse health consequences could be averted. We aimed to investigate the association between serum 25-hydroxyvitamin D (25-OH-D), the major circulating form of VD, and cognitive function, as well as school performance in a high-income setting with abundant sunshine. 

## 2. Methods

This school-based cross-sectional study was conducted in public middle schools selected from the State of Kuwait, a small country with a population of 4.2 million. The study population was students between the ages of 11 and 16 years, who were selected using stratified multistage cluster random sampling, with probability proportional to the size of the schools, from all governorates of Kuwait. In this sampling method, schools with a large number of students were given higher probability to be selected compared to schools with a small number of students. Blood samples were collected in February, March, and April 2016. The study was approved by The Ethics Committee at the Ministry of Health, Kuwait (No: 2015/248) as well as The Ethics Committee of the Health Sciences Centre, Kuwait University (No: DR/EC/2338).

Details of the study have been described previously [8]. In brief, an informed consent form was sent to the parents along with a self-administered questionnaire on various demographic variables and dietary habits. After obtaining written informed consent from the parents and verbal assent from the child, a trained interviewer conducted a face-to-face interview with the child using a structured questionnaire. The questionnaire was carefully developed after extensive review of the literature and was pilot tested on 20 students who were not included in the study. It comprised of questions on habitual sun exposure during the last three months, which was assessed by the core questions developed to measure the exposure to sunlight in adolescents as described by Glanz et al. (2008) [27]; in addition to smoking habits and dietary intake. Dietary intake was evaluated using a food frequency questionnaire for calcium and vitamin D intake in adolescents [28], which has been validated before in our region [29]. Data on physical activity were collected using a questionnaire that was developed based on the Youth Physical Activity Questionnaire in UK [30] and The Arab teens lifestyle study [31]. The questionnaire was validated among high school students and showed a strong correlation with data collected by accelerometers (Spearman’s correlation 0.92; *p* < 0.001 for total steps count; unpublished data). Measurements of standing height and body weight of the study subjects were assessed using digital weight and height scale (Detecto^®^, Webb City, MO, USA) in a standardized manner.

### 2.1. Cognitive Function Tests and Students’ Academic Performance

We used the Raven’s Standard Progressive Matrices (SPM) for assessing the cognitive function of the study participants. SPM is a language and culture-free test, which measures abstract reasoning, fluid intelligence, problem solving, perceptual awareness, and reasoning by analogy. In this test, participants are asked to identify the missing piece of an array of patterns by determining which rule or rules govern the patterns displayed in the rows and columns. The test has been validated in our setting previously [32], and is routinely used by the Ministry of Education, Kuwait, in schools for measuring cognitive function. The test was conducted in groups by trained staff from the Ministry of Education. Raw scores were converted into age-adjusted percentile rank and standard scores as per the instruction manual [33]. The age-adjusted standard score (ASC) was used as the outcome variable. With respect to academic performance, the results of the participants were obtained from the school records. A percentage score obtained by students’ in the exams of Mathematics, Science, and English, which were conducted close to the date of blood collection, were recorded. We also recoded the overall percentage score that the students earned in all exams (school performance), which included other study subjects such as physical education and Arabic literature. School performance (SP) was also used as the outcome variable.

### 2.2. Blood Collection and Biochemical Analyses

Five mL of venous blood was collected from each child and were analyzed for glucose, complete blood count, iron profile, folate, vitamin B_12_, calcium, parathyroid hormone (PTH), and VD. All the blood tests, except plasma 25-OH-D, were conducted in a major secondary care hospital where these tests are routinely performed, with strict quality control measures. Samples for 25-OH-D analyses were protected from light throughout handling and processing. Plasma was separated and stored at −80 °C until analysis. Plasma 25-OH-D was measured in a CAP-accredited laboratory by liquid chromatography-tandem mass spectrometry (LC-MS/MS), as described previously [34].

### 2.3. Statistical Methods

**Figure 1:**
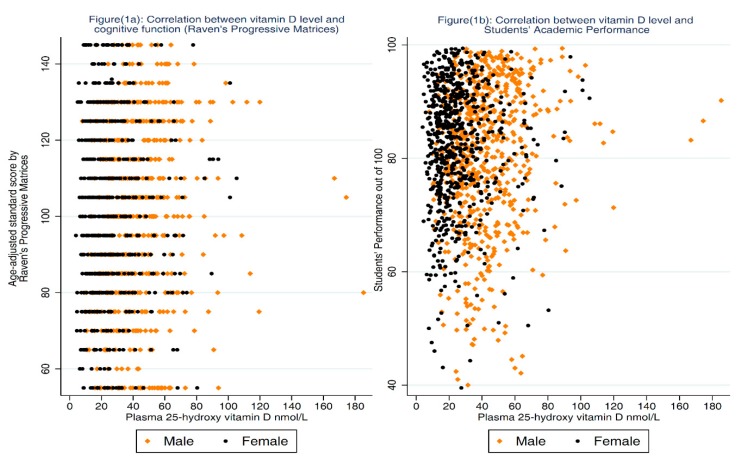
Association between plasma 25-hydroxy vitamin D and age-adjusted standard score (Raven’s Standard Progressive Matrices test) as well as Students’ Academic Performance.

**Figure 2:**
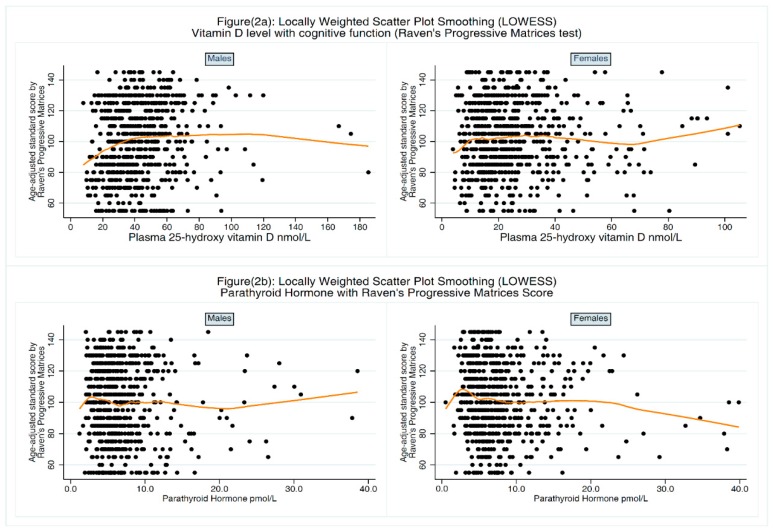
Association between plasma 25-hydroxy vitamin D or parathyroid hormone and age-adjusted standard score (Raven’s Standard Progressive Matrices test).

Data were double entered into specifically designed database using Epidata Entry. Data analysis was conducted using Stata version 12 (StataCorp., College Station, TX, USA). The main outcomes in the analysis were the ASC and the SP. The distribution of ASC and 25-OH-D was evaluated using histograms and normal plots. Spearman’s correlation coefficient (ρ) and scatter plots were used to assess the crude association between ASC and 25-OH-D after stratification by gender (Figure 1a). The same analyses were repeated for the SP as an outcome variable (Figure 1b). We also evaluated the relationship between 25-OH-D level and ASC using LOcally WEighted Scatterplot Smoothing (LOWESS) [35] after stratification by gender (Figure 2a). Similarly, we used this technique to evaluate the relationship between PTH and ASC (Figure 2b). Several statistical approaches, including linear regression, quantile regression and ordered logistic regression, were used to investigate the association between 25-OH-D level and ASC before and after adjusting for potential confounders. During each analysis, 25-OH-D was treated as a continuous variable and then as a categorical variable categorized first by clinically acceptable cutoff points (25-OH-D < 25 nmol/L severe deficiency, 25-OH-D ≥ 25 nmol/L and <50 nmol/L deficiency, 25-OH-D ≥ 50 nmol/L and <75 nmol/L insufficiency, and 25-OH-D ≥ 75 nmol/L sufficiency [36,37]), and second as quartiles.

Covariates were divided into four groups. Using multiple linear regressions, the association between each variable in each group and ASC was assessed and the variables that showed association at a 15% level of significance were included in the multivariate analysis. Each group of covariates was introduced to the model sequentially and the impact of this on the association between 25-OH-D and ASC was noted. The findings from this analysis were replicated using bootstrap estimates. 

**Figure 3:**
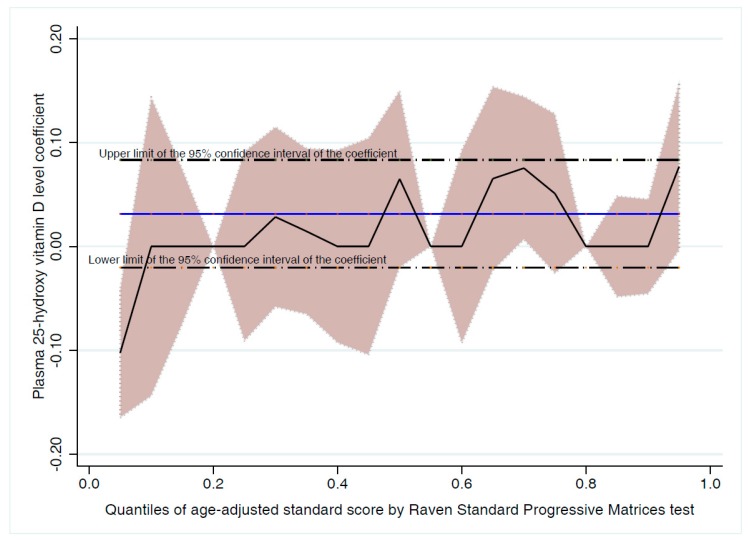
Association between plasma 25-hydroxy vitamin D level and age-adjusted standard score (Raven’s Standard Progressive Matrices test) at different level of cognitive function.

To investigate whether the strength of association between 25-OH-D and cognitive function is different across ASC scores, we repeated the analysis above using simultaneous-quantile regression. This was also a good approach to check whether the slight deviation from the assumptions of the linear regression may give a different conclusion. Quantile regression did not provide significantly different results from multiple linear regression (Figure 3). While all the analysis above used ASC as a continuous variable, we repeated the analysis using ordered logistic regression after categorization of ASC into tertiles. The results of quantile regression and ordered logistic regression analyses were reported in the text as a sensitivity analysis. In all analyses above, we looked for an interaction between gender and 25-OH-D. A similar approach was used to investigate the association between SP and 25-OH-D level. Students’ score in each study subject were negatively skewed and thus we used simultaneous-quantile regression with quantile 0.5 to adjust for the various group of confounders. In this technique, standard errors were obtained via bootstrapping after 1000 replicates. 

## 3. Results

We approached 1583 parents, of which 161 (child, parents, or both) refused to participate. Six blood samples were not sufficient to conduct blood analysis. Of the remaining (*N* = 1416), 1370 adolescents had the test of cognitive function; and their socio-demographic characteristics are shown in Table 1. The median (inter-quartile range, IQR) of 25-OH-D level was 29.7 (19.2–44.1) nmol/L. The median (IQR) of 25-OH-D was 39.8 (29.4–52.7) nmol/L and 21.5 (14.7–30.7) nmol/L among males and females, respectively (*p* < 0.001). The mean (SD) of ASC was 101.3 (23.4), which was not significantly different by gender (*p* = 0.12).

### 3.1. Association between 25-OH-D and Cognitive Function

As shown in Figure 1, a very weak positive correlation was found between the 25-OH-D level and ASC (ρ = 0.06; *p* = 0.04) which became evident among males after stratification by gender (ρ = 0.14; *p* < 0.01 and ρ = 0.03; *p* = 0.43 for males and females respectively). Univariable linear regression analysis showed an association between ASC and 25-OH-D quartiles only after stratification by gender but adjusting for parental education was sufficient to explain this association. The association between 25-OH-D level and ASC in each gender with the LOWESS line is shown in Figure 2a. Using multiple regression, no significant association was found between 25-OH-D level and ASC before and after adjusting for confounders (Table 2). Because PTH can be considered as a proxy indicator for VD status, we investigated the association between PTH and ASC. There was negative weak but statically significant correlation between ASC and PTH (ρ = −0.08; *p* < 0.01). The association between PTH level and ASC in each gender with the LOWESS line is shown in Figure 2b. Regression analysis showed that adjusting for other variables, particularly parental education, was sufficient to explain this association.

We used a stepwise approach to confirm our findings in which 25-OH-D (continuous or categorical) was not selected in any model. When we used the same technique while 25-OH-D was a forced-in variable, it was not significant in any model. With the same technique, however, PTH was negatively associated with ASC when fitted as a continuous variable but not as a categorical variable (β = −0.26; *p* = 0.048 forward selection; β = −0.22; *p* = 0.08 backward selection).

To check whether the slight deviation from the assumptions of the regression analysis may affect our conclusion, we repeated the analysis above using bootstrap methods and quantile regression. The results of both methods mirrored the findings of the linear regression analysis and showed no association between VD level and ASC. 

### 3.2. Association between 25-OH-D and Academic Performance

There was a significant correlation between ASC and SP (ρ = 0.43; *p* < 0.01). Also, there was a very weak but significant correlation between SP and 25-OH-D levels (ρ = 0.05; *p* = 0.045). Quantile regression showed no significant association between 25-OH-D level and SP (Table 3). We repeated the analysis with SP in each study subject including Mathematics, Science, and English separately. There was no significant association between 25-OH-D levels and the SP in any study subject (data not shown). 

## 4. Discussion

Whereas the presence of VDR and VD metabolizing enzymes in several brain areas that are involved in learning and memory in both humans and animals is well documented [15,16,17,18], epidemiological studies on humans have not demonstrated a clear association between VD and cognitive function, particularly among children or adolescents. Using a large nationally representative sample of adolescents, we found no association between 25-OH-D and ASC after adjusting for potential confounders. Our results are consistent with the two studies that used NHANES III data, and showed no association between 25-OH-D and cognitive function [23] or any of the psychometric measures [24]. One study did report lower scores on the Wechsler Intelligence Scale in subjects with VD deficiency compared to non-deficient subjects; however, this study suffered from several methodological weaknesses including a small sample size and inappropriate study design, statistical analysis, and data interpretation [38].

We measured cognitive function using a language and culture-free test (Raven’s SPM) to minimize the influence of language and culture. SPM is the most widely used non-verbal test of cognitive ability and has been reported to provide more comprehensive information on cognitive performance, even in children with cerebral palsy [39]. SPM is a test of abstract reasoning, fluid intelligence, problem solving, perceptual awareness, and reasoning by analogy. In our study, the ASC were highly correlated with students’ performance in mathematics (ρ = 0.45; *p* < 0.01), science (ρ = 0.34; *p* < 0.01), and the overall SP (ρ = 0.43; *p* < 0.01), which increased our confidence in the test. 25-OH-D showed no significant association with students’ overall score or with score in any individual study subject. This is similar to a previous study, which showed that 25-OH-D concentrations for the ages 7.6–11.8 years were not associated with any educational outcomes at the ages 13–14 or 15–16 years [40].

Low serum 25-OH-D is usually associated with high circulating PTH level, a condition known as secondary hyperparathyroidism. In addition to the classical target organs of PTH, kidneys, and bones, PTH receptors are also expressed in the mammalian brain [41]. Furthermore, PTH can cross the blood brain barrier and thus may affect cognitive function independent of VD [42]. We reported a significant association between PTH and 25-OH-D [8]; and therefore tested the association between PTH and cognitive function. When a stepwise approach was used to select variables related to ASC, PTH as a continuous variable was selected in forward selection (*p* = 0.048) but not in backward selection (*p* = 0.08). This statistical method of mechanical selection of variables has been heavily criticized in the literature [43]; therefore, this finding may not warrant further discussion. It is worth noting that no association was found when PTH was categorized either as quartiles or by clinical cutoff points (PTH ≥ 65 ng/L). In adults, elevated serum PTH has been reported to be associated with poor cognitive function [44,45]. To our knowledge, no data is available on the association between elevated serum PTH and cognitive function in children or adolescents. It is recommended that future studies on the association between VD and cognitive function in children consider PTH levels as the effect of VD may be confounded by high serum PTH levels.

Evidence from observational studies suggest that VD is related to cognitive function among older adults [22] but to date there is no single study that has demonstrated this association among adolescents suggesting that this relationship might be age-dependent. Together these studies suggest that VD is more important in preventing cognitive decline, which is associated with older age. If VD impacts cognitive function in a neuroprotective way, then it is understandable to see the association among older adults at the time when cognitive function starts to deteriorate and not during adolescence or young adulthood. Recent literature supports this notion, showing VD deficient individuals experience a faster rate of cognitive decline [46,47]. It is also possible that VD interacts with other factors that associate with aging, hence preserving cognitive function. If this hypothesis is well-founded, then it is logical to investigate the association between VD and cognition at an older age (>65 years) rather than in adolescence [23]. It is also possible that VD has an impact on cognitive function throughout the lifespan with a critical window for VD deficiency during pregnancy and early childhood, and only after a long latency period does its impact become more apparent at older ages when cognitive function starts to decline. This goes with the developmental origin of health and disease (DOHaD) paradigm and is supported by studies that showed the link between VD deficiency during pregnancy and various outcomes during childhood or adulthood [48]. Evidence from animal studies suggest that maternal VD during pregnancy plays a role in the brain development of offspring; however, the association between low maternal VD levels during pregnancy with poor cognition of offspring in humans is inconclusive [49]. The question whether high VD levels reduce the risk of cognitive decline in the elderly or affect cognitive function according to the DOHaD paradigm will determine whether VD supplementation or other preventive measures would be more important during early life or during late adulthood. It is possible that VD may affect cognitive function during both early life as well as late adulthood.

The apparent lack of association between 25-OH-D and cognitive function in our study could also be because of a very high prevalence of VD deficiency/insufficiency in our study population (<4% were VD sufficient). It is well known that epidemiological studies are inefficient in detecting association when the population is universally exposed [50]. In communities where VD deficiency is universally common, large, well-conducted RCTs are warranted to investigate whether VD supplementation would improve the cognitive function of adolescents who have VD deficiency. With RCTs, unlike observational studies, it is possible to create a meaningfully unexposed group (VD sufficient) through VD supplementation that can be compared with a group of adolescents with deficient or insufficient VD. Furthermore, it has been previously suggested that children with better cognitive function may spend more time indoors on scholarly activities, such as reading, compared to children with poor cognitive function, and consequently, have lower levels of VD concentration. Therefore, even if higher VD levels improve cognitive function, such a reverse and inverse relationship may potentially conceal the impact of VD level on cognitive function, if any [23]. Finally, we used the Raven’s SPM and scholarly achievement (school performance) as measures of cognitive function. Although SPM is the most widely used test of cognitive function, it is not the only measure of all cognitive abilities. SPM is a non-verbal test of general cognitive ability but does not measure other cognitive functions such as verbal cognition, non-verbal cognition, executive functioning, and processing speed, among others. It is possible that VD deficiency may be associated with other cognitive abilities in adolescents, which are not measured by the SPM. 

### Strengths and Limitations

This is the first study that investigated the association between VD levels and cognitive function among adolescence in a country with abundant sunshine. The other two studies are based on data from the U.S. (NHANES III). We have adjusted for factors, which other studies have failed to adjust for, including season of birth and sleeping hours, in addition to socio-economic and biochemical factors. We used various statistical techniques to look for the association dealing with VD as a continuous and a categorical variable. We also measured VD using the recommended laboratory technique (LC-MS/MS) [51] compared to other less accurate laboratory methods such as chemiluminescent immunoassay, radioimmunoassay and enzyme-linked immunosorbent assay [52,53]. A limitation of this study is that a single measure of cognition (Raven’s SPM) was used which does not measure all cognitive abilities. Also, we measured 25-OH-D on a single time point, which may not reflect each participant’s long-term VD status. This is likely to attenuate the association between 25-OH-D and ASC if it exists (non-differential misclassification). Finally, early life exposure to inadequate VD levels in utero and/or during infancy was not taken into account.

## 5. Conclusions

In conclusion, only two studies exist on the association between VD and cognitive function in adolescents and both are based on data from the U.S. (NHANES III). This is the first study of this kind on a population outside North America. Our study showed no association between VD levels and cognitive function as measured by ASC (Raven’s SPM) and academic performance, and our findings are consistent with these studies. The fact that several studies demonstrated the relationship between VD and cognitive function among the elderly but not among adolescents suggest that the link between VD and cognitive function may be age-dependent. Further longitudinal studies are warranted to investigate this issue because it has significant implications on VD supplementation. 

## Figures and Tables

**Table 1:** Socio-demographic characteristics of 1370 adolescents in public middle schools in Kuwait.

Characteristics		
**Age in years**, Mean (SD) years	12.4	(0.9)
	*n*	(%)
**Gender**		
Male	674	(49.2)
**Nationality**		
Kuwaiti	1047	(76.4)
Non-Kuwait	323	(23.6)
**Father’s Education** ^1^		
No formal education	15	(1.11)
Primary/Intermediate	215	(16.1)
Secondary (high school)	330	(24.7)
Diploma	251	(18.8)
University & above	526	(39.3)
**Mother’s Education** ^2^		
No formal education	31	(2.3)
Primary/Intermediate	145	(10.7)
Secondary (high school)	294	(21.9)
Diploma	293	(21.7)
University & above	587	(43.5)
**Father’s Income** ^3^ **(Kuwaiti Dinars)**		
Less than 500	89	(6.7)
500 to 1000	291	(22.0)
1001 to 1500	414	(31.3)
1501 to 2000	213	(16.1)
More than 2000	164	(12.4)
Do not wish to tell	153	(11.6)
**Mother’s Employment Status** ^4^		
Housewife	466	(34.7)
Paid employment	664	(49.5)
Others	212	(15.8)
**Housing** ^5^		
Rented flat	499	(36.9)
Rented house	159	(11.8)
Owned flat	55	(4.1)
Owned house	638	(47.2)

^1^ Missing for 33 participants; ^2^ Missing for 20 participants; ^3^ Missing for 46 participants; ^4^ Missing for 28 participants; ^5^ Missing for 19 participants.

**Table 2:** Association between plasma 25-hydroxy vitamin D and the age-adjusted standard score (Raven’s Standard Progressive Matrices test) before and after adjusting for potential confounders.

Vitamin D Status	Model 1	Model 2	Model 3	Model 4	Model 5
	β [95%CI]	β [95%CI]	β [95%CI]	β [95%CI]	β [95%CI]
25-OH-D levels nmol/L	0.03	0.01	0.00	0.00	−0.01
	[−0.02, 0.08]	[−0.04, 0.07]	[−0.05, 0.06]	[−0.05, 0.06]	[−0.07, 0.05]
***p*-value**	0.23	0.70	0.95	0.85	0.73
**Q1** (25-OH-D < 19.2 nmol/L) (*n* = 337)	[Reference]	[Reference]	[Reference]	[Reference]	[Reference]
**Q2** (25-OH-D ≥ 19.2 to <29.7 nmol/L) (*n* = 346)	0.86	1.12	−0.39	1.15	0.82
	[−2.65, 4.38]	[−2.32, 4.58]	[−4.28, 3.49]	[−2.29, 4.59]	[−2.82, 4.46]
**Q3** (25-OH-D from 29.7 to <44.1 nmol/L) (*n* = 344)	2.67	2.29	−0.26	2.69	0.84
	[−0.86, 6.19]	[−1.43, 6.02]	[−6.25−5.72]	[−1.03, 6.41]	[−3.24, 4.92]
**Q4** (25-OH-D ≥ 44.1 nmol/L) (*n* = 343)	2.91	0.95	−0.70	0.50	−0.42
	[−0.62, 6.43]	[−3.00, 4.90]	[−7.92, 6.53]	[−3.47, 4.47]	[−4.82, 4.01]
***p*-value**	0.30	0.67	1.00	0.45	0.88
**Severe deficiency** (25-OH-D < 25 nmol/L) (*n* = 544)	[Reference]	[Reference]	[Reference]	[Reference]	[Reference]
**Deficiency** (25-OH-D ≥ 25 to <50 nmol/L) (*n* = 572)	2.28	2.52	2.70	2.71	1.36
	[−0.46, 5.04]	[−0.39, 5.44]	[0.22, 5.63]	[−0.22, 5.63]	[−1.86, 4.59]
**Insufficiency** (25-OH-D ≥ 50 to <75 nmol/L) (*n* = 205)	1.40	−0.66	−0.93	−0.94	−1.53
	[−2.36, 5.16]	[−4.66, 3.34]	[−4.94, 3.09]	[−4.97, 3.08]	[−6.01, 2.94]
**Sufficiency** (25-OH-D ≥ 75 nmol/L) (*n* = 49)	5.46	4.98	2.79	3.02	1.47
	[−1.39, 12.31]	[−1.96, 11.92]	[−4.16, 9.74]	[−3.93, 9.98]	[−5.96, 8.91]
***p*-value**	0.24	0.11	0.11	0.10	0.48

Q1 to Q4: quartile one to quartile four; Model 1: unadjusted; Model 2: adjusted for socio-demographic factors (gender, nationality, governorate, education of the father, education of the mother, type of housing, season of birth, and passive smoking at home); Model 3: adjusted for variables in Model 2 in addition to grade level, sleeping hours during weekends, having nap during weekend, time spent outside during the last 3 months during weekdays and weekends, reporting medical condition, and frequency of consumption of sugary drinks; Model 4: adjusted for variables in Model 3 in addition to BMI categories; Model 5: adjusted for variables in Model 4 in addition to parathyroid hormone, vitamin B_12_, and transferrin saturation all as quartiles.

**Table 3:** Association between plasma 25-hydroxy vitamin D and school performance before and after adjusting for potential confounders (Median Regression).

Vitamin D Status	Model 1	Model 2	Model 3	Model 4	Model 5
	β [95%CI]	β [95%CI]	β [95%CI]	β [95%CI]	β [95%CI]
25-OH-D levels nmol/L	0.02	−0.00	−0.00	−0.01	−0.01
	[−0.01, 0.06]	[−0.03, 0.03]	[−0.04, 0.03]	[−0.04, 0.03]	[−0.04, 0.03]
***p*-value**	0.22	0.94	0.77	0.67	0.69
**Q1** (25-OH-D < 19.2 nmol/L) (*n* = 337)	[Reference]	[Reference]	[Reference]	[Reference]	[Reference]
**Q2** (25-OH-D ≥19.2 to <29.7 nmol/L) (*n* = 346)	1.10	1.61	2.01	1.66	1.74
	[−1.54, 3.74]	[−0.34, 3.57]	[0.01, 4.01]	[−0.42, 3.75]	[−0.33, 3.80]
**Q3** (25-OH-D from 29.7 to <44.1 nmol/L) (*n* = 344)	0.40	0.70	0.98	0.90	0.94
	[−2.15, 2.95]	[−1.58, 2.99]	[−1.26, 3.21]	[−1.39, 3.20]	[−1.41, 3.30]
**Q4** (25-OH-D ≥ 44.1 nmol/L) (*n* = 343)	2.60	0.82	1.00	1.40	1.67
	[0.06, 5.25]	[−1.51, 3.14]	[−1.38, 3.38]	[−1.03, 3.83]	[−0.86, 4.19]
***p*-value**	0.17	0.43	0.27	0.45	0.36
**Severe deficiency** (25-OH-D < 25 nmol/L) (*n* = 544)	[Reference]	[Reference]	[Reference]	[Reference]	[Reference]
**Deficiency** (25-OH-D ≥ 25 to <50 nmol/L) (*n* = 572)	−0.30	−1.36	−0.84	−0.99	−0.94
	[−2.35, 1.75]	[−3.04, 0.32]	[−2.65, 0.96]	[−2.73, 0.73]	[−2.88, 0.99]
**Insufficiency** (25-OH-D ≥ 50 to <75 nmol/L) (*n* = 205)	2.30	−0.34	0.31	0.28	0.38
	[−0.33, 4.93]	[−2.46, 1.77]	[−2.51, 1.88]	[−1.89, 2.47]	[−1.97, 2.73]
**Sufficiency** (25-OH-D ≥ 75 nmol/L) (*n* = 49)	3.10	−1.71	−1.27	−2.12	−1.99
	[−0.83, 7.03]	[−5.15, 1.73]	[−5.39, 2.84]	[−5.99, 1.74]	[−5.86, 1.89]
***p*-value**	0.08	0.38	0.78	0.35	0.45

Q1 to Q4: quartile one to quartile four; Model 1: unadjusted; Model 2: adjusted for socio-demographic factors (age, gender, nationality, season of birth, governorate, education of the father, education of the mother, total number of siblings, father income, employment of the mother, type of housing, and child birth order), passive smoking at home, frequency of having breakfast before going to school, and frequency in addition to the number of times the child had breakfast or dinner prepared outside their home; Model 3: adjusted for variables in Model 2 in addition to sleeping hours during weekends having nap during weekend, time of the first meal during weekend and weekdays, and frequency of sugary drinks; Model 4: adjusted for variables in Model 3 in addition to BMI categories; Model 5: adjusted for variables in Model 4 in addition to parathyroid hormone.

## Abbreviation

25-OH-D	25-hydroxy vitamin D
ASC	age-adjusted standard score
CAP	College of American Pathologists
LC-MS/MS	liquid chromatography tandem mass spectrometry
PTH	parathyroid hormone
RCT	randomized control trials
SP	school performance
SPM	Standard Progressive Matrices
VD	vitamin D
VDR	vitamin D receptors
